# Balancing competition for resources with multiple pest regulation in diversified agroecosystems: a process‐based approach to reconcile diversification and productivity

**DOI:** 10.1002/ece3.2453

**Published:** 2016-11-11

**Authors:** Charlotte Poeydebat, Dominique Carval, Luc de Lapeyre de Bellaire, Philippe Tixier

**Affiliations:** ^1^ CIRADCAECLe Lamentin Cedex 2MartiniqueFrance; ^2^ UPR 26 GECOCIRADMontpellier Cedex 5France; ^3^ Departamento de Agricultura y AgroforesteriaCATIECartagoTurrialbaCosta Rica

**Keywords:** crop model, injury profile, plant trait, resource balance, vegetation assemblage, yield losses

## Abstract

Agroecosystem plant diversification can enhance pest biological regulation and is a promising alternative to pesticide application. However, the costs of competition for resources between plants may exceed the benefits gained by pest regulation. To disentangle the interactions between pest regulation and competition, we developed a generic process‐based approach that accounts for the effects of an associated plant and leaf and root pests on biomass production. We considered three crop–plant associations that differ in competition profiles, and we simulated biomass production under wide ranges of both pest regulation rates and resources’ availability. We analyzed outputs to quantify the pest regulation service level that would be required to attain monoculture yield and other production goals. Results showed that pest regulation requirements were highly dependent on the profile of resource interception of the associated plant and on resources’ availability. Pest regulation and the magnitude of competition between plants interacted in determining the balance between nitrogen and radiation uptake by the crop. Our findings suggest that productivity of diversified agroecosystems relative to monoculture should be optimized by assembling plants whose characteristics balance crops’ resource acquisition. The theoretical insights from our study draw generic rules for vegetation assemblage to optimize trade‐offs between pest regulation and production. Our findings and approach may have implications in understanding, theorizing and implementing agroecosystem diversification. By its generic and adaptable structure, our approach should be useful for studying the effects of diversification in many agroecosystems.

## 1 Introduction

According to the resource concentration hypothesis (Root, 1973), intensive cropping systems, in which crops are cultivated at high densities in large fields, are prone to pest infestation. In such systems, crop protection and yield rely on pesticides that can threaten biodiversity and human health (Aktar, Sengupta, & Chowdhury, 2009; Tilman, Cassman, Matson, Naylor, & Polasky, 2002). To be more sustainable, but still efficient in limiting yield losses, pest management should be based on a systemic approach that accounts for multiple pests and that combines biopesticides, biological control agents, pheromones, ecological engineering of plant biodiversity, and cultural practices (Birch, Begg, & Squire, 2011; Lewis, Van Lenteren, Phatak, & Tumlinson Iii, 1997). In this sense, agroecosystem plant diversification is increasingly considered a promising way to restore ecosystem functions, including ecological pest regulation (Altieri, 1999; Gurr, Wratten, & Luna, 2003; Leakey, 2014; Malézieux et al., 2009; Tscharntke et al., 2012).

Plant diversification alters the properties of agroecosystems in terms of both resource partitioning (Malézieux et al., 2009) and pest regulation (Letourneau et al., 2011). On the one hand, field‐scale diversification often results in yield losses (Letourneau et al., 2011; Quijas, Schmid, & Balvanera, 2010) because of competition for resources between the crop and associated plants. The magnitude of this competition depends on the availability of resources and on the functional and architectural complementarity of plant traits involved in resource capture (Brooker et al., 2015; Roscher et al., 2012; Zuppinger‐Dingley et al., 2014). On the other hand, plant diversification may interfere with pest regulation by affecting life cycles and dispersion of populations of pests and agents of biological control and their interactions through modifications of (1) the microclimate; (2) the diversity and concentration of resources; (3) the diversity and fragmentation of habitats; and (4) the chemical environment (Altieri & Letourneau, 1982; Norris & Kogan, 2005; Ratnadass, Fernandes, Avelino, & Habib, 2012; Schroth, Krauss, Gasparotto, & Duarte, 2000; Trenbath, 1993). Agroecosystem plant diversification at the field scale has apparently enhanced ecological pest regulation in many cases and for diverse pests (Letourneau et al., 2011; Quijas et al., 2010). In some situations, however, plant diversification can favor pests (Norris & Kogan, 2005; Schroth et al., 2000) and can reduce pest regulation and increase pest damage (Letourneau et al., 2011; Quijas et al., 2010).

Because most pests damage crop organs involved in resource acquisition, pest regulation, and resource partitioning strongly interact to determine crop growth and yield. Consequently, the increased ecological pest regulation gained from plant diversification may be outweighed by a stronger competition for resources. To optimize crop biomass production when introducing associated plants in an agroecosystem, yield losses induced by competition for resources between the crop and associated plants should be compensated by yield gains resulting from higher pest regulation.

Insights into the effects of plant diversification on production, pest regulation, and other ecosystem services have been obtained by combining experimental studies with statistical models (Bradford et al., 2014; Poveda, Martínez, Kersch‐Becker, Bonilla, & Tscharntke, 2012) and by meta‐analyses (Iverson et al., 2014; Letourneau et al., 2011). Unlike process‐based approaches, these methods allow little extrapolation, prediction, or clarification of the underlying processes. Schipanski et al. (2014) assessed various ecosystem services using process‐based modeling but were forced to use semiquantitative estimates of pest regulation, based on the literature and expert knowledge, because of a lack of an appropriate simulation tool. Although process‐based models have been used to assess the effect of pest management strategies on pest dynamics and crop performance, authors have not included plant diversification as a strategy and thus ignored the potential effects of plant competition (Grechi et al., 2012; Lô‐Pelzer et al., 2010). Other authors designed models simulating the effect of plant diversity on crop production through competition without including pests (Brisson, Bussière, Ozier‐Lafontaine, Tournebize, & Sinoquet, 2004; Munier‐Jolain, Guyot, & Colbach, 2013; Schipanski et al., 2014; Shili‐Touzi, De Tourdonnet, Launay, & Dore, 2010). To our knowledge, no process‐based model has been specifically developed to disentangle the interactions between competition for resources and pest regulation at crop scale in a general plant diversification perspective.

In this study, we present a process‐based agroecosystem modeling approach that combines a validated crop growth model with the impact of an associated plant and two pest types on crop's resource uptake. We simulate three archetypal scenarios involving associated plants with different profiles of resource interception under wide ranges of resources’ availability. We quantified pest regulation rates required to compensate for yield losses due to competition, compared to monoculture yield and a range of production goals, depending on the profile of resource interception of the associated plant and resources’ availability. We analyzed model outputs to gain theoretical and generic knowledge about crop–plant–pest interactions in diversified agroecosystems.

## 2 Methods

### 2.1 Process‐based approach

**Figure 1:**
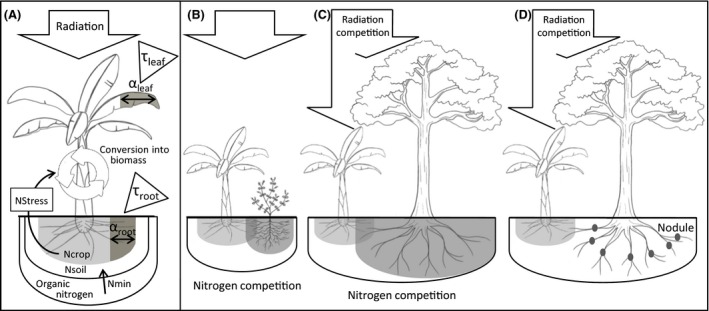
Schematic diagram of the modeling framework (A) and of the scenarios of diversification (B–D). (A) In the crop model, leaf area index and root biomass are damaged by pests following α_leaf_ and α_root_ rates, respectively. Leaf and root damages are regulated according to τ_leaf_ and τ_root_ rates, respectively. Organic nitrogen is mineralized at Nmin rate and added to the stock of soil mineral nitrogen. The part of Nsoil accessible to the crop, Ncrop, depends on the functional root biomass. When Ncrop fall below a threshold, crop growth is affected by nitrogen stress, NStress. Three plants were associated with the crop for simulation: (B) a ground plant competing for nitrogen only, (C) a nonleguminous tree competing for nitrogen and radiation (D), and a leguminous tree competing for radiation only

From previous parametrized and calibrated modeling works (Ripoche, Achard, Laurens, & Tixier, 2012; Tixier, Malézieux, Dorel, & Wery, 2008), we developed a simplified model structure for the simulation of diversified agroecosystems that combine a crop model with profiles of pest injury and profiles of resource interception by an associated plant (Fig. 1). This model simulated crop biomass on a weekly time step. Three crop phenological stages were distinguished and successively triggered according to heat‐unit accumulation thresholds. Biomass was allocated to the different parts of the crop according to the stage. Vegetative (leaves, roots, and pseudostem) and reproductive (fruit bunch) biomass were expressed as kg plant^−1^ year^−1^. The global incident radiation (GRad), intercepted by the crop was proportional to the crop's leaf area index and was converted into biomass according to a radiation‐use efficiency coefficient. The mineral nitrogen content of the soil (Nsoil), depended on the initial stock value and on a constant nitrogen mineralization rate (Nmin), and reflected overall soil fertility. Crop nitrogen uptake was deducted from Nsoil at each time step *t*. The amount of nitrogen available to the crop (Ncrop), was proportional to Nsoil but was also determined by the ratio between the actual root biomass at *t* and the potential root biomass that can be attained under optimal growth conditions. When Ncrop was below 38 kg N/ha, the crop was considered to suffer from nitrogen stress whose intensity increased with Ncrop decrease (Ripoche et al., 2012). This stress affected crop growth by reducing heat‐unit accumulation and biomass production. Water was considered to be nonlimiting for crop growth.

Additionally to crop growth simulation, our model accounted for the effects of pests and an associated plant on crop growth. Pests were classified into root and leaf pest types, and each type was characterized by the proportion of organ damaged at each time step, α_root_ and α_leaf,_ respectively. Pest damage rates were constant across simulations and throughout the crop cycle. Damages reduced functional biomass and thus the crop's ability to use nitrogen and radiation resources. Pest regulation rates, τ_root_ and τ_leaf_, respectively, for root and leaf pests, that were constant throughout the crop cycle, were applied to pest damage rates to reduce pest damages. At each simulation time step, crop biomass production was penalized by the proportion of crop organs destroyed by the pests depending of the final damage rates resulting from the product between pest damage and regulation rates. The banana tree was considered to develop in an intermediary height stratum, while the associated plant was standing either below or above the crop. The relative height of the crop and the associated plant were constant throughout the crop cycle. We assumed that the associated plant had a constant biomass and was characterized by its light radiation interception coefficient, β_radiation_, and its nitrogen demand, β_nitrogen_. β_radiation_ values depended on the height of the plant relative to that of the crop, and β_nitrogen_ values depended on the plant's ability to fix atmospheric nitrogen; both coefficients were constant throughout the crop cycle. At each time step *t*, nitrogen uptake by the associated plant was deducted from Ncrop.

Details and R code of the framework are provided in Appendix 1 in Supporting Information.

### 2.2 Application to virtual scenarios of banana agroecosystem diversification

**Figure 2:**
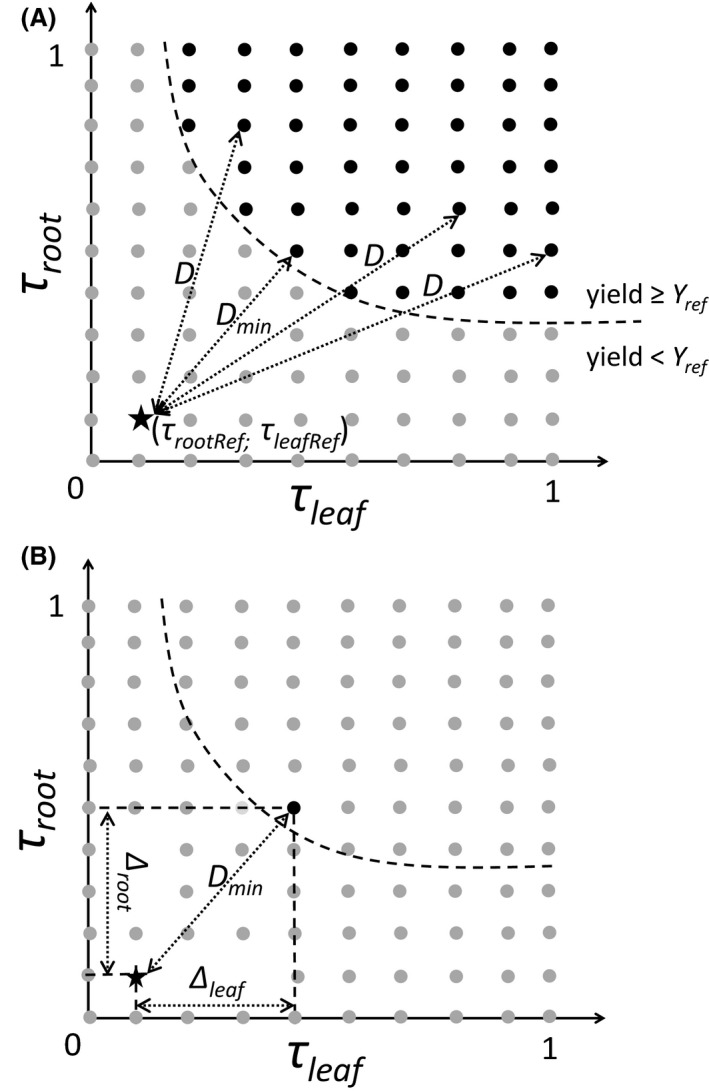
Calculation of the minimal pest regulation effort (MPRE) needed to attain the monoculture yield, *Y*_ref_. The procedure was repeated for each diversification scenario in each resource context. In each case, the yield of the diversification scenario and *Y*_ref_ against which it was compared were resulting from the same resource context. (A) Black dots represented all the combinations of leaf and root pest regulations rates, τ_leaf_ and τ_root_, respectively, enabling the attainment of or exceeding of *Y*_ref_. The black star corresponded to the leaf and root pest regulation rates applied to the monoculture scenario, τ_leafRef_ and τ_rootRef_ *,* respectively. Euclidean distances *D* between each black dot and the black star were calculated. (B) The minimal *D* value, *D*_min_, was considered to be the MPRE. ∆_leaf_ and ∆_root_ are the values of leaf and root pest regulation efforts required to attain *Y*_ref_ corresponding to *D*_min_

The framework presented above may be used for any crop for which a parametrized and validated growth simulation model is available. Here, we used a banana agroecosystem as the model system because: (1) previous modeling work provided us with a calibrated banana crop model (Ripoche et al., 2012; Tixier et al., 2008); (2) banana plants have an intermediate position in the canopy, which is essential for studying interspecific competition for light radiation; and (3) the wet tropical conditions under which bananas are grown allowed us to assume that weather was constant and to avoid needing climatic data. To illustrate the relationship between production and pest regulation in diversified agroecosystems, we simulated three virtual scenarios of diversification in which the banana crop was associated with a plant with one of three resource interception profiles: (1) a ground plant (GP) standing below the crop and competing for nitrogen only (Fig. 1B); (2) a tree (T) standing above the crop and competing for radiation and soil nitrogen (Fig. 1C); and (3) a nitrogen‐fixing tree (NFT), competing only for radiation (Fig. 1D). The coefficients of resource interception describing the profile of the associated plant in terms of competition were set arbitrarily to represent the different scenarios. Parameters from the crop model were calibrated from previous works (Table 1). Air temperature was set to be representative of tropical conditions and assumed to be constant. Nmin and GRad varied to represent the ranges of nitrogen and radiation availability likely to be encountered in fields.

**Table 1:** Values and references for calibration of model parameters

Parameter	Value	Description	References for calibration
STFini (degree days)	1,400	Thermal time sum from planting to flowering initiation	
STFlo (degree days)	400	Thermal time sum from flowering initiation to flowering	
STFH (degree days)	900	Thermal time sum from flowering to harvest	Tixier (2004)
*T*_0_ (°C)	14	Basal temperature for development	Tixier (2004)
Ea	0.95	Photosynthetically active radiation	
Ec	0.48	Photosynthetically active radiation intercepted	Ripoche et al. (2012)
Eb	0.018	Conversion efficiency	
*K*	0.7	Crop coefficient	Nyombi et al. (2009)
FWC	0.75	Fruit/bunch water content	
seneBF	0.017	Rate of leaf senescence before flowering	Ripoche et al. (2012)
seneAF	0.025	Rate of leaf senescence after flowering	Ripoche et al. (2012)
SLA (m²/kg dry leaf)	7.4	Specific leaf area (leaf surface by biomass unit)	Ripoche et al. (2012)
LFpcent (%)	0.34	Percent of assimilates allocated to leaf during vegetative growth	Ripoche et al. (2012)
S (m²)	5.3	Ground surface of the banana tree	Ripoche et al. (2012)
Rootmax^a^ (kg)	1.75	Potential root biomass	
RTpcent	0.22	Percent of vegetative biomass allocated to the roots	Tixier (2004)
TNcrop (% N)	0.008	Banana tissue nitrogen content	
Nthreshold (kg N/ha)	38	Soil nitrogen content threshold below which stress can occur	Ripoche et al. (2012)
α_leaf_	0.08	Leaf necrosis rate induced by pest	
τ_leaf_ b	0–1	Regulation rate of leaf pest damage	
α_root_	0.05	Root necrosis rate induced by pest	
τ_root_ b	0–1	Regulation rate of root pest damage	
β_radiation_ c (%)	15	Percent of radiation intercepted by the associated plant when shading	
β_nitrogen_ c (kg N ha^−1^ week^−1^)	2	Nitrogen demand of the associated plant when nonleguminous	
GRad^b^ (MJ m^−2^ day^−1^)	9–15	Daily global radiation	
Temp (°C)	25	Air temperature	
Nmin^b^ (kg N ha^−1^ week^−1^)	0–6	Soil nitrogen mineralization rate	
Nsoil (Kg N/ha)	100	Initial stock of soil nitrogen	

a Rootmax value was obtained by simulating crop growth under potential growth conditions.

b The numbers in the “value” column correspond to the extreme values of the range used in the study.

c Parameters corresponding to resource interception by the associated plant. Indicated values correspond to cases where the associated plant competes with the crop for the resource. When the associated plant does not compete for a resource, the corresponding parameter is set to 0.

The model was deliberately based on a series of assumptions, that is, no water limitation, constant weather, constant associated plant biomass and resource interception, constant relative heights of crop and associated plant, constant pest damage and regulation rates. This approach aimed at limiting the number of varying parameters and variables to provide simpler and clearer interpretations of modeling outputs while staying representative of real conditions, such as perennial systems or systems with regenerating GP cover under tropical conditions.

### 2.3 Quantifying pest regulation service that would compensate for competition in diversified agroecosystems

From a general point of view, our objective was to simulate crop yield under a set of growth situations determined by resources’ availability, resources’ interception by an associated plant, and leaf and root pest damage (both resulting from the product of pest damage rate and pest regulation rate). We simulated all the possible combinations of variable values, as in a sensitivity analysis design, and then studied the combination of input and output variables to explore the relationship between yields, competition for resources and pest regulation.

More precisely, we first propose a generic procedure to quantify the minimal pest regulation effort (MPRE) required to compensate for yield losses in plant‐diversified agroecosystems, as depending on resource competition and availability. The reference scenario against which the yield (bunch weight) and pest regulation rates of the diversification scenarios were compared was a banana monoculture in which pest regulation rates τ_rootRef_ and τ_leafRef_ were set at 0.2. The yield of this reference scenario, *Y*_ref_, was simulated along a gradient of Nmin, while radiation was set at a median level. Crop yield of each diversification scenario (bunch weight) was also simulated along the Nmin gradient with a median radiation level and for all combinations of values of τ_root_ and τ_leaf_ ranging from 0 when regulation was nil to 1 when regulation was complete. For a diversification scenario and resource level, we selected the combinations of τ_leaf_ and τ_root_ values that allowed crop yield to be greater or equal to *Y*_ref_. In such situations, pest regulation rates induced yield gains that fully compensated for the yield losses due to competition. We calculated the Euclidean distance *D* between each selected combination and the combination of pest regulation rates of the reference scenario (Fig. 2A). We considered the minimal *D* value, *D*_min_, to be the MPRE required to compensate for yield losses due to competition (Fig. 2B). *Δ*_root_, the difference between τ_rootRef_ and τ_root_, and Δ_leaf_, the difference between τ_leafRef_ and τ_leaf_, represented the root and leaf pest regulation efforts corresponding to the MPRE, respectively. We repeated this procedure along a gradient of light radiation, GRad.

### 2.4 Sensitivity of crop yield to pest regulation in diversified agroecosystems

To broaden our analysis, we determined a series of production goals ranging from 0 to 45 kg bunch weight plant^−1^ year^−1^ and we explored the sensitivity of the yield to pest regulation under three contrasted levels of resource availability (low, intermediate, and high) and for each scenario of diversification. For each diversification scenario and resource level, we plotted one isocline per production goal corresponding to pairs of root and leaf pest regulation rates that allowed the attainment of the production goal. The procedure was reproduced for radiation and nitrogen resources.

## 3 Results

### 3.1 Quantifying pest regulation service that would compensate for competition in diversified agroecosystems

**Figure 3:**
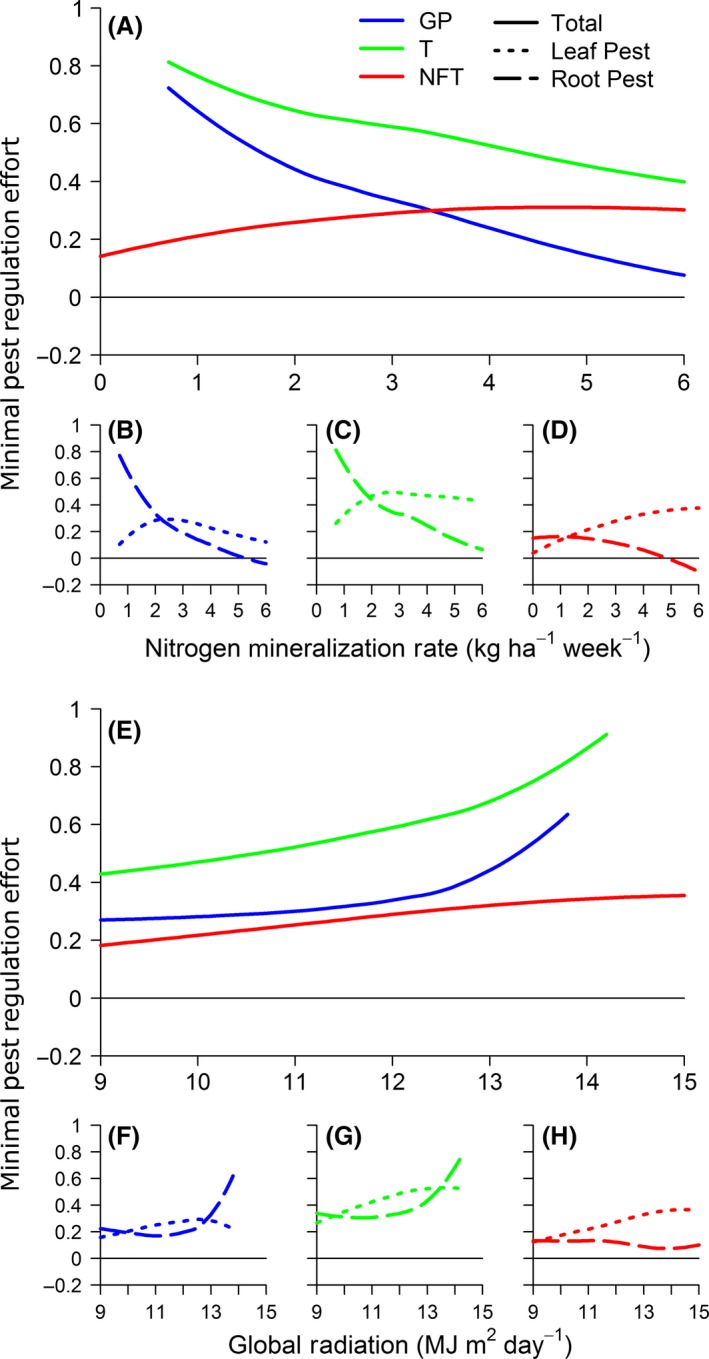
Minimal pest regulation effort (MPRE) needed to compensate for yield losses due to competition. MPRE is computed for three diversification scenarios: when a ground plant (GP), a tree (T), or a nitrogen‐fixing tree (NFT) is introduced as an associated plant, and along a gradient of nitrogen mineralization rate (Nmin) (A) and a gradient of radiation (E). The regulation effort required for leaf pest and root pest is plotted using dotted lines and dashed lines, respectively, for the three diversification scenarios and for the Nmin gradient (B–D) and the radiation gradient (F–H)

Figure 3A shows the MPRE needed to attain *Y*_ref_ as a function of Nmin for three diversification scenarios involving associated plants with different profiles of resource interception: a GP, a tree (T), and a leguminous tree (NFT). For GP and T scenarios, *Y*_ref_ was impossible to achieve for the lowest Nmin values, and the overall MPRE decreased as Nmin increased. For low Nmin values, this decrease was related to the strong decrease of root pest regulation effort, which exceeded the increase in leaf pest regulation effort (Fig. 3B, C). For higher Nmin values, regulation efforts of both pests decreased. Inversely, for the NFT scenario, MPRE increased along the Nmin gradient, which was entirely related to the increase in leaf pest regulation effort (Fig. 3D), whereas root pest regulation effort decreased. MPRE was minimal for NFT at low‐to‐intermediate Nmin values but was minimal for GP at higher Nmin values. MPRE was always positive but the root pest regulation was negative for the highest Nmin values in GP and NFT.

Minimal pest regulation effort (MPRE) increased with incident radiation, GRad, in all diversification scenarios (Fig. 3E). It was always lower for NFT than for the other two scenarios. From low‐to‐intermediate GRad values, MPRE increased slightly in T and NFT and even more slightly in GP due to an increase in leaf pest regulation effort. Above intermediate GRad values, MPRE increased more in T and GP. This abrupt increase was related to increased root pest regulation effort, whereas leaf pest regulation effort decreased (Fig. 3F, G). Although MPRE had the same pattern in GP and T, it was lower in GP than in T. For the highest range of GRad values, *Y*_ref_ was not attainable in GP and T even with complete pest regulation (Fig. 3E). In NFT, MPRE increased constantly along the entire radiation gradient. In this scenario, the increase of MPRE mainly resulted from increased leaf pest regulation effort (Fig. 3H). Pest regulation efforts were always positive.

### 3.2 Sensitivity of crop yield to pest regulation in diversified agroecosystems

**Figure 4:**
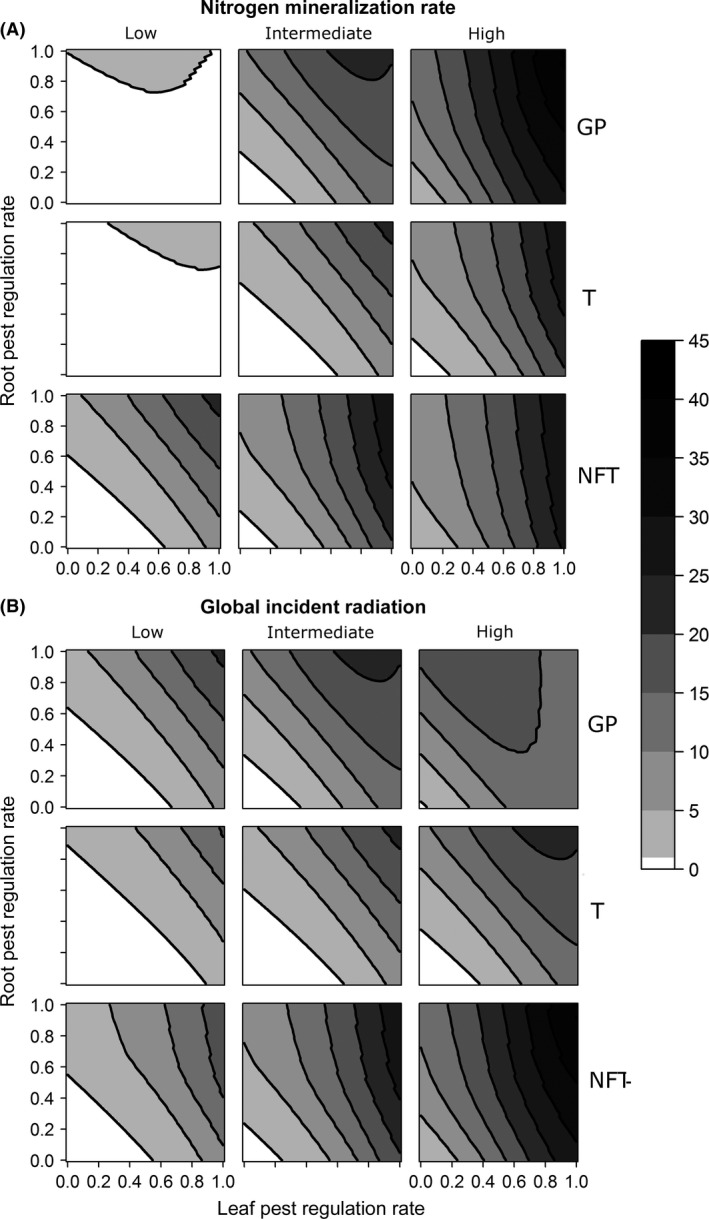
Isoclines of leaf and root pest regulation rates to attain production goals in associations. Pest regulation rates allow compensating for yield losses due to the association of the crop with plant having different profile of competition for resources: a ground plant (GP), a tree (T), or a nitrogen‐fixing tree (NFT). They are reported for low, intermediate, and high nitrogen mineralization rates (Nmins) combined to intermediate radiation level (A) and for low, intermediate, and high radiation levels combined to intermediate Nmin (B). The scale on the right describes production goals expressed in kg plant^−1^ year^−1^

In a given context, different combinations of pest regulation rates may lead to the same production goal (Fig. 4A, B). In most cases, it was impossible to simultaneously minimize regulation rates for leaf and root pests. Negative slopes of isoclines indicated that if one pest regulation rate decreased, the production goal could be maintained by an increase in the other pest regulation rate. The steepness of the slope of the production isoclines demonstrated the relative importance of leaf and root pest regulation and the relative sensitivity of yield to both rates. Steeper slopes suggested that the yield was more sensitive to regulation of leaf pests than root pests. The distance between the production isoclines provided insight on the sensitivity of the yield to overall pest regulation.

In all scenarios, the steepness of the isoclines increased with Nmin, indicating a reinforcement of the relatively higher sensitivity of yield to leaf pest regulation as nitrogen availability increased (Fig. 4A). Under a low Nmin for GP and T and under an intermediate Nmin for GP only, isoclines were concave for the highest attainable production goals, indicating that above a given leaf pest regulation rate, high production goals were maintained because of a joint increase in both pest regulation rates. For all scenarios, the highest production goal attainable increased with the Nmin. With low and intermediate Nmins, the highest production goals were attained in NFT. With a high Nmin, the highest production goals were attained in GP. GP and T performed similarly in terms of highest attainable production goal across Nmins, but pest regulation rates were always higher in the T scenario for a given production goal under a given Nmin.

Because the steepness of the isoclines was constant and almost equal to −1, crop yield was constantly and equally sensitive to root and leaf pest regulation in GP and T regardless of radiation level. In NFT, isoclines steepness was always higher than in the other scenarios and slightly increased with radiation level suggesting that the higher sensitivity of yield to leaf pest regulation was reinforced as radiation availability increased (Fig. 4B). Under a high radiation level in GP and T and under an intermediate radiation level in GP only, the isoclines of the highest production goals were concave, indicating that above a given leaf pest regulation rate, production goals were maintained because of a joint increase in both pest regulation rates. Generally, the overall pest regulation required to attain a given production goal decreased as the radiation level increased. For a given level of radiation, the pest regulation rates corresponding to a given production goal were always highest in T. GP and T performed almost identically in terms of the pest regulation rates required to attain the highest production goals, and NFT always allowed the attainment of higher production goals than the two other scenarios.

## 4 Discussion

The combination of very low fertility and a nonleguminous associated plant may lead to early and strong nitrogen stress because of additive effects of nitrogen deficiency and competition. In such situations, the crop never attains the monoculture yield even with complete pest regulation. With low fertility, however, an associated leguminous tree leads to relatively high crop yields for relatively low pest regulation rates. Consistent with the concept of niche differentiation (MacArthur & Levins, 1967), the drastically lower root pest regulation rate required with a leguminous tree indicated that complementarity in profiles of nitrogen capture between the associated plant and the crop results in reduced competition for nitrogen. This phenomenon has been reported in many intercropping systems involving legumes (Brooker et al., 2015). In addition to complementary nitrogen use, trees may induce facilitation in nitrogen‐poor environments by improving radiation regulation and the nutrient status of the understory crop (Isaac, Ulzen‐Appiah, Timmer, & Quashie‐Sam, 2007). Facilitation based on a reduction in resource disparity has been confirmed to enhance resource use efficiency and crop performance (Garcia‐Barros & Ong, 2004). In nitrogen‐poor environments, complementarity or facilitation between plants may limit the need for pest regulation service provision. As fertility improves, however, the effect of competition for nitrogen on yield decreases, and the advantage of a leguminous versus a nonleguminous associated plant declines. When fertility is high, high leaf pest regulation or nonshading conditions are required to boost radiation conversion in order to support the high crop growth potential provided by nitrogen. These results confirm the prediction that, in agroforests, the benefit of soil fertility improvement through mulch, or avoided competition in the case of the leguminous tree, is greater with low than with high fertility where the negative effects of shading dominate (van Noordwijk, 1996).

Regardless of the profile of resource interception of the associated plant, the increase in radiation availability results in a counter‐intuitive requirement for higher pest regulation. With a leguminous tree where only radiation conversion limits growth, pest regulation increases slightly and is exclusively related to leaf pest regulation. With a nonleguminous associated plant, there is a threshold in the balance between both resources that induces a shift in pest regulation requirements. Below this threshold, although the root pest regulation effort also contributes importantly to overall pest regulation requirements, the increase in pest regulation is related to leaf pest in order to limit the nitrogen stress reinforcement induced by growing radiation conversion. Above the threshold, the increase in radiation reverses the balance between resources and induces a drastic demand for root pest regulation. The leguminous tree minimizes pest regulation requirements regardless of the level of radiation because shading limits radiation conversion and because competition for nitrogen is absent. In the other scenarios, the benefit of higher radiation conversion allowed by higher radiation availability is outweighed by its negative effect on crop growth because of increased nitrogen stress due to higher crop nitrogen demand. This result confirms the findings of Isaac et al. (2007), who suggested that the benefits of radiation reduction could be canceled when the shading tree competes for soil resources.

Given a particular level of resource availability and a particular associated plant profile, various combinations of the regulation rate of the two pests can lead to the attainment of targeted production goals. In most cases, the regulation of one pest may compensate for damage from the other. It means that, generally, the productivity of diversified agroecosystems can be optimized through vegetation characteristics providing either a strong control of one of the two pests or a medium control of both pests. However, when nitrogen availability is poor relative to radiation and when the production goal is high, both pest regulation rates are positively correlated and no longer compensate for each other. Instead, increased leaf pest regulation improves crop radiation conversion to the point where nitrogen may become limiting and this amplification of the disparity in resource supplies combined to a high production goal leads to an increase of the need for root pest regulation. Although our interest is in ecological pest regulation, these results may already be of particular importance to limit superfluous costs and pollution related to chemical or mechanical pest regulation. When nitrogen is nonlimiting, the range in pest regulation rates that allowed the attainment of a given production goal is high and narrow for the leaf pest while it could range from 0 to 1 for the root pest. This indicates that, when nitrogen is less limiting than radiation, crop yield is more sensitive to leaf pest than to root pest regulation and that radiation conversion limitation prevails in yield losses. Moreover, in such conditions, high levels of leaf pest regulation but reduced levels of root pest regulation are required to attain yield equivalent to the monoculture. In contrast, when nitrogen is the most‐limiting factor, yield losses are compensated for by a high root pest regulation rate along with a low leaf pest regulation rate that also contributes in reducing crop demand for nitrogen and therefore nitrogen stress. Consequently, the relative sensitivity of crop yield to leaf or root pest regulation depends on the magnitude and direction of resource imbalance. Depending on resource conditions, improving yield of diversified agroecosystems will be easier by increasing regulation rate of one of the two pests preferentially.

Pest regulation requirements were highly dependent on the profile of resource interception of the plant and resources’ availability. We showed that pest regulation and the magnitude of competition between plants interact in determining the balance between nitrogen and radiation uptake by the crop. Incorporating resource gradients in our study allowed us to detect and quantify the strong dependency of crop–plant–pest interactions on resource balance. Our findings suggest that productivity of diversified agroecosystems relative to monocultures should be optimized by assembling plants whose characteristics balance crop resource acquisition. This conclusion is consistent with Schroth et al. (2000), who suggested that diversified agroecosystems should be designed to reduce the disparity in resource supply and crop stress. Similar to growth stimulation that favors the organ that captures the most‐limiting resource (Bloom, Chapin, & Mooney, 1985), we found that pest regulation was most beneficial when it protected the organ that captures the most‐limiting resource. Under some resource conditions, however, pest regulation should not only consist of reducing damage to organs involved in most‐limiting resource acquisition but also in leaving damage to organs involved in nonlimiting resource acquisition.

Explicit simulation process‐based models were recently used to assess the effect of diversity on the stability and productivity of forests (Morin, Fahse, de Mazancourt, Scherer‐Lorenzen, & Bugmann, 2014) and to assess the effect of outbreak severity on tree biomass while considering various pest damage pathways (Dietze & Matthes, 2014). The theoretical knowledge from our study demonstrates the value of such process‐based integrative tools and contributes to a process‐based understanding of the general relationship between ecosystem diversity and function. Turnbull, Levine, Loreau, and Hector (2013) and related studies have focused on within‐trophic level interactions to explain the effect of diversity on ecosystem functioning, with an emphasis on coexistence and productivity in plant communities. They demonstrated that the difference in productivity between a mixture and equivalent monocultures, the “net biodiversity effect,” results from selection and complementarity effects (Loreau & Hector, 2001) that depend on fitness and niche interspecific differences, respectively (Turnbull et al., 2013). Although we provide a more static representation of the plant community (we assumed a stable community and no fitness difference between the crop and the associated plant), our framework was nevertheless able to reproduce community‐level interactions. For instance, the impossibility of achieving the yield of the monoculture when introducing an associated plant in some circumstances in our study reflects competitive exclusion. Most importantly, our results show that pest regulation may mitigate the effect of competition for resources between plants on crop yield, particularly in resource‐limited environments. We suggest that the role of indirect plant–plant interactions involving multiple trophic levels or abiotic factors, such as pest regulation, should be integrated into frameworks that attempt to explain ecological community outcomes.

Our simple, process‐based approach relied on the hybridization of a validated crop model with functional profiles of pests and associated plants. Functional profiles of plants have been shown to accurately describe resource partitioning and aboveground biomass production in complex agroecosystems (Collalti et al., 2014; Damour, Dorel, Quoc, Meynard, & Risède, 2014; Roscher et al., 2012). We also used pest functional groups depending on the crop organ affected, as has been successfully performed by previous authors (Dietze & Matthes, 2014; Willocquet et al., 2002). Because of its hybrid structure, our model embraces the complexity of diversified agroecosystems and bridges the gap between process‐based and functional‐trait approaches. In addition, the functional traits used to describe competition between plants may also be involved in pest regulation. For instance, height, which is useful to characterize radiation competition in a plant community (Kunstler et al., 2016), is related to the ability of plants to control pests (Damour et al., 2014; Schroth et al., 2000). Our approach could easily be adapted into a model including explicit ecological pest regulation pathways relying on vegetation characteristics. More generally, although our study focuses on archetypal situations, the generic and adaptable structure of our model should make it useful for application to a wide range of agroecosystems involving a wide range of pests.

In conclusion, our study provides generic rules for vegetation assemblages that may contribute to the implementation of agroecosystem diversification. It promotes the development of integrative approaches and tools to elucidate the complex interactions between plants, pests, and resources ruling the outcome of diversified agroecosystems. Moreover, our findings suggest that ecological theory concerning plant communities should be expanded to include indirect interactions between plants that may interfere with resource use and fitness of plant species, such as those involving pest regulations. Ultimately, the knowledge and approach presented here may be of valuable support to develop policies or diversified cropping system designs promoting multiple ecosystem services.

## Funding Information

This work was supported by CIRAD and was funded by the Project “Use of the biodiversity of Martinique to improve the functioning of agro‐ecosystems” from E.U. FEDER (grant PRESAGE no. 33157).

## Conflict of Interest

None declared.
