# Supplementary material for: Balancing competition for resources with multiple pest regulation in diversified agroecosystems: a process‐based approach to reconcile diversification and productivity
Source: Ecol Evol. 2016 Nov 11;6(23):8607–16. doi: 10.1002/ece3.2453 (PMC5167016; doi:10.1002/ece3.2453)
Supplement: Supplementary file 1 [file ECE3-6-8607-s001.docx]

**Appendix 1** *Details and R code of the process-based model*

1. Details

We developed a simple, general model representing a multi-strata agroecosystem (AES) in which the crop occupies the intermediate stratum in the canopy and is susceptible to leaf and root pests. This discrete-time model simulates the growth of a single plant with a weekly step *t*. We consider a homogeneous stand, and yield is extended to a 1-ha field considering that each plant occupies a surface *S* of the field. Weather input data include global incident radiation *GRad* and air temperature *Temp*, which are considered constant. Water was not considered as a limiting factor.

The phenology of the crop depends on heat-unit accumulation. The sum of thermal-time ST is calculated at each time step following a temperature base *T0*.

$$ST\left( t \right)=ST\left( t-1 \right)+(Temp\left( t \right)-T0)$$

We distinguish three phenological stages: the vegetative growth stage, the flowering stage, and the post-flowering stage. These stages are triggered according to different ST thresholds (see Table 1) and determine the allocation of biomass to the different plant parts. During the vegetative growth stage, the rate of biomass allocated to the vegetative parts, *VGrate*, is equal to 1, meaning that all biomass is allocated to vegetative parts. No biomass is allocated to reproductive parts, so that the rate of biomass allocated to reproductive parts, *RPrate*, is equal to 0. Leaves, stem, and roots receive *LFpcent*, *STpcent*, and *RTpcent* percent, respectively, of the biomass allocated to the vegetative parts (see Table 1). During the flowering stage, biomass is distributed between vegetative and reproductive parts following *RPrate*, which is calculated as:

$$RPrate\left( t \right)= \frac{\left( ST\left( t \right)-STFini \right)}{STFlo}$$

where *STFini* and *STFlo* are the thermal-time sum thresholds that trigger flowering initiation and the post-flowering stage, respectively. During the post-flowering stage, all biomass produced is allocated to reproductive parts, with *RPrate* equal to 1 and *VGrate* equal to 0. Harvest was triggered when the heat-units required to complete the post-flowering stage were accumulated.

The crop grows as a function of radiation and nitrogen. Crop interception of radiation is proportional to its leaf area:

$$PARi\left( t \right)=Ea\times Ec\times GRad\times\left( 1-e{}^{(-K\times LAI\left( t-1 \right))} \right)$$

where *Ea* is the percentage of photosynthetically active radiation, *Ec* is the percentage of photosynthetically active radiation intercepted by the crop, *GRad* is the weekly global incident radiation, *K* is the crop coefficient, and *LAI* is the leaf area index. At each time step *t*, biomass accumulated, *∆biom(t)*, is calculated as a function of *PARi*:

$$\Delta biom\left( t \right)=Eb\times PARi\left( t \right)$$

where *Eb* is the light-to-biomass conversion efficiency of the crop. *LAI* is then calculated as a function of *∆biom(t)* as follows:

$$LAI\left( t \right)=LAI\left( t-1 \right)+\left( \frac{\Delta biom\left( t \right)\times SLA\times VGrate\times LFpcent}{S} \right)-\left( LAI\left( t-1 \right)\times SENrate \right)$$

where *SLA* is the specific leaf area, and *SENrate* is the rate of leaf senescence, which varies with the stage of development (see seneBF and seneAF in Table 1).

The root biomass at time step *t* was calculated as a fraction *RTpcent* of the biomass allocated to vegetative parts:

$$BiomRT \left( t \right)=BiomRT\left( t-1 \right)+\left( \Delta biom\left( t \right)\times VGrate\times RTpcent \right)$$

The initial stock of soil nitrogen, *Nsoil*, is supplied throughout the crop cycle by a constant nitrogen mineralization rate, *Nmin*. At each time step *t*, the stock of soil nitrogen, *Nsoil*, is calculated depending on *Nmin* and the amount of nitrogen removed by the crop, *CropUptake*, as follows:

$$Nsoil\left( t \right)=Nsoil\left( t-1 \right)+Nmin-CropUptake$$

and $CropUptake =\left( \frac{\Delta biom\times TNcrop}{S} \right)\times10000$

where *TNcrop* is the nitrogen content of the crop. Nitrogen available to the crop, *Ncrop*, was determined by the percentage of root growth completed, *RTG*:

$$Ncrop\left( t \right)=Nsoil\left( t \right) \times RTG$$

with $RTG\left( t \right)=\frac{BiomRT\left( t \right)}{Rootmax}$

*Rootmax* is the potential root biomass that can be attained under optimal growth conditions. Because roots are initiated only during the pre-flowering stage ([Gousseland & Lavigne 1984](#_ENREF_1); [Lavigne 1987](#_ENREF_2)), we considered that the root biomass is maximal at flowering.

We consider that a nitrogen stress, *NStress(t)*, can occur when *Ncrop(t)* falls below a threshold, *Nthreshold* ([Ripoche et al. 2012](#_ENREF_3)).

$$NStress\left( t \right)=\frac{Ncrop(t)}{Nthreshold}$$

This stress affects heat-unit accumulation and biomass production as follows:

$$ST\left( t \right)=\left( ST\left( t-1 \right)+(Temp\left( t \right)-T0) \right)\times NStress(t)$$

$$\Delta biom\left( t \right)=Eb\times PARi\left( t \right)\times NStress(t)$$

Crop growth can be affected by the action of two pests. One pest damages leaves and reduces the functional *LAI* of the crop with a damage rate of *α_leaf_*. The other pest damages roots and reduces *Ncrop* with a damage rate of *α_root_*. For both pests, the damage rate was constant during the whole crop cycle. The effects of leaf and root pests on the crop were decreased by constant regulation rates, *τ_leaf_* and *τ_root_*, respectively (see Table 1). In the case of pest damage occurrence, *LAI(t)* and *BiomRT(t)* equations were modified as follows:

$$LAI\left( t \right)=LAI\left( t-1 \right)+\left( \frac{\Delta biom\left( t \right)\times SLA\times VGrate\times LFpcent}{S} \right)-\left( LAIh\times SENrate \right)-(LAI(t-1)\times\alpha\text{l}\text{eaf})$$

where *LAIh* is the healthy leaf area of the crop, i.e., excluding parts affected by pest necrosis, and is calculated as follow:

$$LAIh=LAI\left( t-1 \right)\times(1-\alpha\text{l}\text{eaf})$$

$$BiomRT \left( t \right)=BiomRT\left( t-1 \right)+\left( \Delta biom\left( t \right)\times VGrate\times RTpcent \right)-(BiomRT\left( t-1 \right) \times\alpha\text{r}\text{oot}\text{ })$$

Pest damage rates were possibly adjusted through pest regulation coefficients τ*_leaf_* and τ*_root_*, which were applied as follow:

$$\alpha\text{l}\text{eaf}= \alpha\text{l}\text{eaf}\times\left( 1- \tau\text{l}\text{eaf} \right)$$

$$\alpha\text{r}\text{oot}= \alpha\text{r}\text{oot} \times(1- \tau\text{r}\text{oot})$$

The structure of the model enables us to add an associated plant in the simulated AES. We consider that this associated plant has a constant biomass and is characterized by its light interception coefficient, *β_radiation_*, and its nitrogen demand per week, *β_nitrogen_*; *β_radiation_* depends on the height of the plant relative to that of the crop, and *β_nitrogen_* depends on the plant’s ability to fix nitrogen. The profile of resource interception of the associated plant determines the degree of competition between the crop and the associated plant. In the presence of an associated plant in the AES, *GRad(t)* and *Nsoil (t)* were modified as follows:

$$GRad\left( t \right)=GRad(t)\times(1-\text{β}\text{radiation}\text{ })$$

$$Nsoil\left( t \right)=Nsoil\left( t-1 \right)+Nmin-\beta_{nitrogen}- CropUptake$$

1. R Code

#********************************************************************************#

# Setting the range of values for resource gradients, pest regulation rates & associated plant profiles

#********************************************************************************#

# Nitrogen Mineralization Rate Gradient

input = expand.grid (tauLeaf = seq (0, 1, by=0.02), tauRoot = seq (0, 1, by=0.02), betaLight = c (0, 0.15), betaNitrogen = c (0, 2), Nsoil = 100, Nmin = seq (0, 6, by=0.1), GRad = 12)

# Global Radiation Gradient

input = expand.grid (tauLeaf = seq (0, 1, by=0.02), tauRoot = seq (0, 1, by=0.02), betaLight = c (0, 0.15), betaNitrogen = c (0, 2), Nsoil = 100, Nmin = 3, GRad = seq (9, 15, by=0.1))

# Profile of associated plants for resources interception

# betaLight = 0 & betaNitrogen = 0 ........................ Monoculture (reference scenario)

# betaLight = 0 & betaNitrogen = 2 ........................ Ground Plant (GP)

# betaLight = 0.15 & betaNitrogen = 0 ................... Nitrogen-fixing Tree (NFT)

# betaLight = 0.15 & betaNitrogen = 2 ................... Tree (T)

#********************************************************************************#

# Set the model constant parameters

#********************************************************************************#

# Temporal duration of simulation in weeks

tmax=100

# Crop growth parameter settings

Ea=0.95

Ec=0.48

Eb=0.018

K=0.7

FWC=0.75

SeneBF=0.017

SeneAF=0.025

NStress=1

Rootmax=1.75

RTpcent=0.22

# Crop architecture parameter settings

SLA=7.4

LFpcent=0.34

S=5.3

# Crop phenology parameter settings

T0=14*7

STFini=1400

STFlo=400

STFH=900

# Crop-nitrogen relationship parameter settings

TNcrop=0.008

Nthreshold=38

# Pest damage rate settings

alphaLeaf=0.08

alphaRoot=0.05

# Climate inputs (temperature in °C)

Temp=25

WeekTemp=Temp*7

# Variables initialization

LAI=0.1

BiomVegetative=0.05

BiomFruit=0

BiomRT=0.3

ST=0

IniF=0

Flowering=0

Harvest=0

HarvWeek=0

CropUptake=0

CropUptakeTot=0

#********************************************************************************#

# Setting the model variables and simulation process

#********************************************************************************#

**for** (i in 1:length(input[,1]))

{

# Creation of a data frame to store the model outputs

outputs=data.frame(t=c(1:tmax) )

# Store the value of varying parameters

tauLeaf=input$tauLeaf[i]

tauRoot=input$tauRoot[i]

betaLight=input$betaLight[i]

betaNitrogen=input$betaNitrogen[i]

Nmin=input$Nmin[i]

Nsoil=input$Nsoil[i]

GRad=input$GRad[i]*7

# Computing actual radiation and pest damage rates (after interception and competition)

GRad=GRad*(1-betaLight)

alphaLeaf=alphaLeaf*(1-tauLeaf)

alphaRoot=alphaRoot*(1-tauRoot)

# Simulation of crop growth

**for** (t in 1:tmax)

**{**

# Nitrogen soil stock

**if**(Nsoil+Nmin-betaNitrogen>=CropUptake)

**{**Nsoil=Nsoil+Nmin-betaNitrogen-CropUptake**}**

**else**

**{**Nsoil=0**}**

# Phenology

**if**(IniF==0)

**{**ST=ST+((WeekTemp-T0)*(NStress))**}**

**else**

**{**ST=ST+(WeekTemp-T0)**}**

**if**(ST>=STFini) (IniF=1) **else** (IniF=0)

**if**(ST>=STFini+STFlo) (Flowering=1) **else** (Flowering=0)

**if**(ST>=(STFini+STFlo+STFH)) (Harvest=1) **else** (Harvest=0)

**if**(IniF==0 & Flowering==0) (VGrate=1)

**if**(IniF==1 & Flowering==0) (VGrate=(1-(ST-STFini)/STFlo))

**if**(IniF==1 & Flowering==1) (VGrate=0)

**if**(Harvest==1 & HarvWeek==0) {HarvWeek=t}

# Light interception

PARi=Ea*Ec*GRad*(1-exp(-K*LAI))

# Light to biomass conversion

deltabiom=Eb*PARi*NStress

# Biomass allocation

BiomVegetative=BiomVegetative+(deltabiom*VGrate)

BiomFruit=BiomFruit+(deltabiom*(1-VGrate))

BiomRT=BiomRT+(deltabiom*RTpcent*VGrate)-(BiomRT*alphaRoot)

RTG=BiomRT/Rootmax

**if** (RTG>1) **{**RTG=1**} else** **{**RTG=RTG**}**

# Crop nitrogen demand

CropDemand=(Eb*PARi)*TNcrop

# Crop nitrogen uptake (multiply by 10 000 to obtain the value for 1 ha field)

CropUptake=((deltabiom*TNcrop)/S)*10000

CropUptakeTot=CropUptakeTot+CropUptake

# Nitrogen stress

NCrop=(Nsoil*RTG)

**if**(NCrop<=Nthreshold & CropDemand>0) **{**NStress=(NCrop/Nthreshold)**}**

**else** {NStress=1}

**if**(t>2) **{**NStress=(NStress+outputs$NStress[t-1])/2**}** **else** (NStress=1)

outputs$NStress[t]=NStress

# LAI formation & senescence

if(Flowering==0) (SENrate=SeneBF) else (SENrate=SeneAF)

LAIh=LAI*(1-alphaLeaf)

LAI=LAI+((deltabiom*LFpcent*SLA*VGrate)/S)-(LAIh*SENrate)-(LAI*alphaLeaf)

**if**(Harvest==1) **{**LAI=0**}**

# Crop Yield

**if**(HarvWeek>0) **{**yield=(((BiomFruit/HarvWeek)*52)/(1-FWC))**} else** **{**yield=0**}**

}

#Save the yield value

input$yield[i]=yield

}

**References**

Gousseland, J. & Lavigne, C. (1984) enracinement et émission racinaire du bananier (Giant Cavendish CV 901) dans les andosols de la Guadeloupe. *Fruits,* **39,** 107-111.

Lavigne, C. (1987) Contribution à l'étude du système racinaire du bananier. Mise au point de rhizotrons et premiers résultats *Fruits,* **42,** 265-271.

Ripoche, A., Achard, R., Laurens, A. & Tixier, P. (2012) Modeling spatial partitioning of light and nitrogen resources in banana cover-cropping systems. *European Journal of Agronomy,* **41,** 81-91.
